# Electromagnetic navigation reduces radiation exposure for retrograde drilling in osteochondrosis dissecans of the talus

**DOI:** 10.1186/s12891-021-04010-4

**Published:** 2021-02-03

**Authors:** Oliver D Jungesblut, Josephine Berger-Groch, Michael Hoffmann, Malte Schroeder, Kara L. Krajewski, Ralf Stuecker, Martin Rupprecht

**Affiliations:** 1 Department of Pediatric Orthopedics, Altonaer Children’s Hospital, Bleickenallee 38, 22763 Hamburg, Germany; 2 Department of Trauma and Orthopaedic Surgery, University Medical Center Hamburg-Eppendorf, Martinistr. 52, 20246 Hamburg, Germany; 3 Department of Trauma-, Orthopaedic Surgery and Sports Medicine , Asklepios Hospital St. Georg , Lohmühlenstr. 5, 20099 Hamburg, Germany

**Keywords:** Electromagnetic navigation, Talus, Retrograde drilling, Osteochondritis dissecans, adolescent, cartilage

## Abstract

**Background:**

Retrograde drilling in osteochondrosis dissecans (OCD) is a widely used surgical intervention. A radiation-free electromagnetic navigation system (ENS)-based method was compared with the standard freehand fluoroscopic (SFF) method regarding clinical applicability.

**Methods:**

We performed a clinical cohort study at a department of Orthopaedics in a Level 1 children’s hospital with 40 patients (20 SFF and 20 ENS). Retrograde drilling of the talar dome was used in patients with unstable medial OCD (MRI stage 2 according to Hepple’s revised classification; stage 2 according to the International Cartilage Repair Society). The outcome measurements were: (a) Intraoperative fluoroscopy exposure and length of surgery and (b) Postoperative serial follow-up MRIs every 6 months.

**Results:**

22 female and 18 male patients aged 13.8 ± 1.6 years (range: 11–17 years) were included. Using the ENS technique, length of surgery was significantly reduced to 20.2 ± 6.4 min compared to 36.1 ± 11.8 min (p < 0.01) for the SFF technique. The average x-ray radiation time for the SFF technique was 23.5 ± 13.5 sec and 1.9 ± 1.7 sec for the ENS technique (*p* < 0.01). Radiation exposure was significantly reduced from 44.6 ± 19.7 mSv (SFF technique) to 5.6 ± 2.8 mSv (ENS technique) (*p* < 0.01). Intraoperative perforation of cartilage occurred once in the SFF group. Correct placement of the drilling channel was verified in all patients on follow-up MRI after six months and a timely healing was seen after two years.

**Conclusions:**

The ENS method provides for a significant reduction in length of surgery and radiation exposure. ENS was without intraoperative cartilage perforation. The clinical and radiological follow-up parameters are comparable for SFF- and ENS-guided retrograde drilling.

**Trial registration:**

WF – 085/20, 05/2020 “retrospectively registered” https://www.aerztekammer-hamburg.org/ethik_kommission.html.

## Background

Increasing participation of children in competitive sports with intensive training [1] as well as increasing obesity in childhood [2], both risk factors for osteochondrosis dissecans (OCD) of the talus, in addition to an ever-increasing use of MRI imaging [3] have led to an increase in the incidence of talar OCD. There is a peak in patients between 12 and 19 years of age [4]. The therapy of talar OCD is challenging and a variety of conservative and surgical methods exist [5]. In contrast to adults, spontaneous healing of talar OCD has been documented in children [6]. In stable OCD, non-surgical treatment includes abstinence of certain sports and compression loads as well as adjustment of vitamin D levels [7, 8]. An efficient and commonly employed surgical therapy of nearly unstable OCD is arthroscopically-assisted drilling for subchondral decompression and revascularization [9–13].

Retrograde drilling avoids articular surface violation in most cases, but control of the drill depth and drill placement can be challenging and require radiographic guidance [14]. As a result of the complexity of these surgeries, numerous approaches have been reported: freehand fluoroscopy-guided drilling with or without guiding techniques [15] such as computed tomography [16, 17], MRI [18, 19] and ultrasound-guided [20] options, as well as opto-electronically [21] and x-ray [22] guidance.

The aim of this study was thus to compare a novel electromagnetic navigation system (ENS)-based technique with the standard freehand fluoroscopically guided procedure in patients with talar OCD. The precision of the drilling with the ENS method was well documented in previous cadaver studies. [16, 23–25] We hypothesized, that (I) the length of operation, (II) the radiation exposure, and (III) the number of intra-operative complications, are reduced with the ENS-technique.

## Methods

In this clinical cohort study, 40 patients were included from March 2014 to October 2016. Inclusion criteria were: (I) the presence of an atraumatic medial lesion in MRI (stage 2 according to Hepple et al. [26] and stage 2 according to the International Cartilage Repair Society [27]), (II) a history of pain for a minimum of six months, (III) age under 18 years at initial presentation, and (IV) written consent by the parents. Patients with previous surgery, lateral lesions, posttraumatic lesions, or those requiring arthrotomy and surgical refixation were excluded. The patients included were alternately treated with either the standard free-hand fluoroscopic (SFF) or the ENS technique. Both techniques were executed only by two senior surgeons with the same level of experience in both techniques. Every surgeon treated half of the children via ENS and half of the children via the SFF technique.

### Operative techniques

In both techniques, a standard arthroscopy of the upper ankle joint was performed. The OCD lesion was identified on the medial talar dome and intra-operative fluoroscopy (Ziehm imaging GmbH, Nürnberg, Germany, type Ziehm Solo) and the indication for retrograde drilling was confirmed in all cases. The indication for retrograde drilling was given when overlying cartilage was seen to be intact during arthroscopy (stage 2 according to the ICRS-OD classification [28]).

#### Retrograde drilling via standard freehand fluoroscopical guidance (SFF- method)

A 1.6mm guide-wire was advanced through the distal lateral side of the talar cortex into the OCD using pulsed fluoroscopical guidance in antero-posterior and lateral views. Once the surgeons identified a distance of 2mm to the joint line, the procedure ended. The final K-wire position was fluoroscopically documented in two planes. Three K-wires were positioned and finally the drilling was executed with a cannulated 2.9mm drill above all three K-wires. At the end of operation, the cartilage surface was checked for perforation via arthroscopy.

#### Retrograde drilling via electromagnetical guidance (ENS-method)

**Fig. 1:**
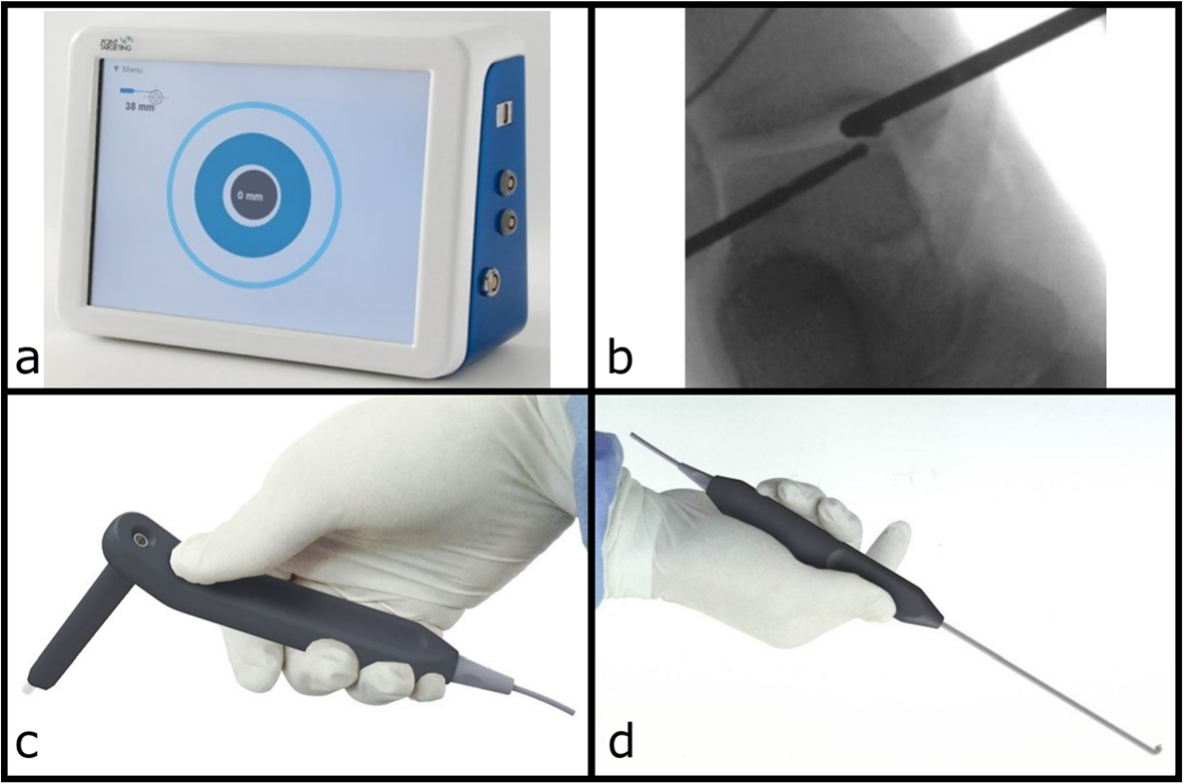
**a**/**b**/**c**/**d** Intraoperative equipment: **a**Monitor. **b** Intraoperative x-ray. **c **Drill sleeve. **d** Probe hook

The NaviDrill electromagnetic targeting device was implemented in this study (NaviDrill™, Arthrex Inc., Naples, FL, USA). Using electromagnetic tracking data obtained intraoperatively, the system provides real-time information on operative instrument placement displayed on a monitor (Fig. 1a). A special probe hook of small size was constructed for ankle joint arthroscopy (Fig. 1b). The drill sleeve contains the electromagnetic field generator. An electromagnetic sensor is implemented into the tip of the probe hook and calibrated once during the fabrication process (Fig. 1c and d). Spatiotemporal referencing is gained from the sensor within the electromagnetic field provided by the drill sleeve. The correct angle and direction are ensured by a continuous visual real-time feedback of the drill position. Pre- or intra-operative calibrations as well as patient reference bases are not required.

**Fig. 2:**
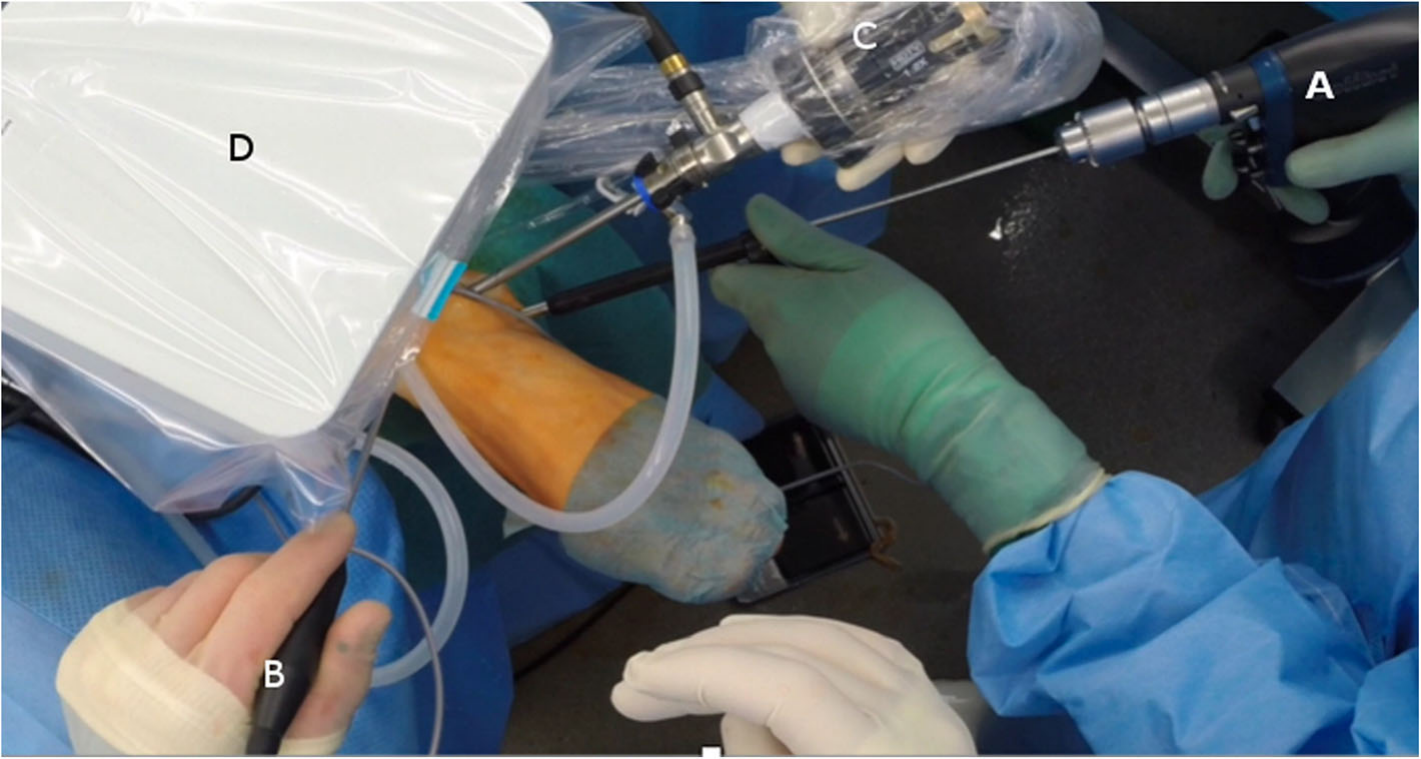
Intraoperative setting; **a** Drill, **b** Probe hook, **c** Arthroscope, **d** x-ray

For this procedure, the probe hook was placed in three different positions within the area of the OCD. The position of the probe hook was continuously monitored via arthroscopy. Similar to the free-hand fluoroscopically assisted technique, a skin incision was made with subsequent blunt preparation to the bone cortex of the talus. A 1.6mm K-wire was passed through a central slot in the electromagnetic field generator (Fig. 2).

The entry point was chosen about 2 cm distal of the lateral standard arthroscopy portal. Subsequent a one-time control by fluoroscopy was performed to preserve the lateral gutter. Additionally, the final position of the k-wire was documented fluoroscopically.

Drilling depth was derived from the information provided on the distance between the tip of the probe hook and the drill sleeve as visualized on the monitor. A stop mark was set on the K-wire using a marker pen (indicated distance minus 2mm to avoid penetrating cartilage) in line with the drilling depth. Real-time drilling direction was provided on the monitor during navigation and allowed for adjustments. As soon as the stop mark on the K-wire was reached, drilling was stopped. After positioning three K-wires, the drilling was executed with a 2.9mm cannulated drill.

### Measurements

We analyzed the radiation exposure and length of surgery for all cases. All 40 patients were seen in our outpatient clinic in a six months rhythm after surgery and underwent magnetic resonance imaging (MRI). Follow-up MRIs were performed in radiology clinics freely chosen by the patients according to our specifications. We required a minimum standard of a 1.5 Tesla MRI generating 3mm fat suppressed layers (T2 coronar, sagittal, and transaxial). The MRIs were analyzed in a systematic and quantitative manner by two independent raters with regard to position, size, and development in sagittal (antero-posterior) and coronal plane (medio-lateral). Raters were not blinded to the diagnosis of OCD but to size and location of the OCD. Results were compared and differences measuring 1mm were rated as equal. In case of differences of ≥ 1mm, the arithmetic mean of both measurements was chosen.

Range of motion and pain on visual analog scale (VAS) were also documented at outpatient visits.

### Statistical analyses

Descriptive statistics were used to describe the basic characteristics of the data set. Continuous variables were presented as a mean and standard deviation (SD). Differences between groups were calculated using the Mann-Whitney-U-Test. A *p*-value < 0.05 was considered statistically significant. Statistical analyses were performed using SPSS statistical software (SPSS version 19.0, Chicago, IL, USA). We calculated Cohen’s kappa coefficients with 95 % confidence interval for all three classification systems. This acts as a measure of the level of agreement among the raters. A value of 1.0 means that there is a perfect agreement among the observers. A value of 0 suggests that the agreement was no better than chance alone. A value of less than 0 suggests greater than random disagreement [29].

## Results

Twenty two female and 18 male patients aged 13.8 ± 1.6 years (range: 11–17 years) were included in the study. 20 patients were treated by the ENS- and 20 patients were treated by the SFF method. The mean MRI size of OCD lesions for in the SFF technique group was 1.14 ± 0.14 cm in the sagittal plane and 0.73 ± 0.12 cm in the coronal plane. Patients undergoing the ENS technique had a lesion measuring 1.16 ± 0.18 cm in the sagittal plane and 0.76 ± 0.11 cm in the coronal plane on MRI (see Table 1). No significant differences (*p*>0.05) regarding size were detected. All lesions were localized on the medial shoulder of the talus. In the ENS technique group, length of surgery was 20.2 ± 6.4 min compared to 36.1 ± 11.8 min in SFF technique group (*p* < 0.01).

**Table 1:** Data set

	SFF technique	ENS technique
Age	13.75 ± 1.74	13.6 ± 1.67
Gender	8 male, 12 female	10 male, 10 female
Localization OCD tali	10 right medial 10 left medial	10 right medial 10 left medial
OCD Size in MRI sagittal (cm) OCD Size in MRI coronar (cm)	1.14 ± 0.14 0.73 ± 0.12	1.16 ± 0.18 0.76 ± 0.11

The average x-ray radiation time in the SFF technique group was 23.5 ± 13.5 sec and 1.9 ± 1.7 sec in the ENS technique group (*p* < 0.01) Radiation exposure was thus reduced from 44.6 ± 19.7 mSv to 5.6 ± 2.8 mSv (*p* < 0.01).

Perforation of cartilage occurred in one case in the SFF technique group (see Table 2).

**Table 2:** Results

	SFF technique	ENS technique	*p*-value
Operation time (min)	36.05 ± 11.75	20.15 ± 6.42	*p* < 0.01
Radiation time (sec)	23.05 ± 13.53	1.90 ± 1.72	*p* < 0.01
Radiation exposure (mSv)	44.58 ± 19.68	5.58 ± 2.76	*p* < 0.01
Complications	1	0	

Six months postoperatively, all patients presented in our outpatient clinic with current MRIs for follow-up. The average time of total follow-up was 30.0 ± 4.3 months. The follow-up MRI scans showed a steadily integration of the cartilage in all cases. The kappa value for interobserver reproducibility of size was 0.9.

**Fig. 3:**
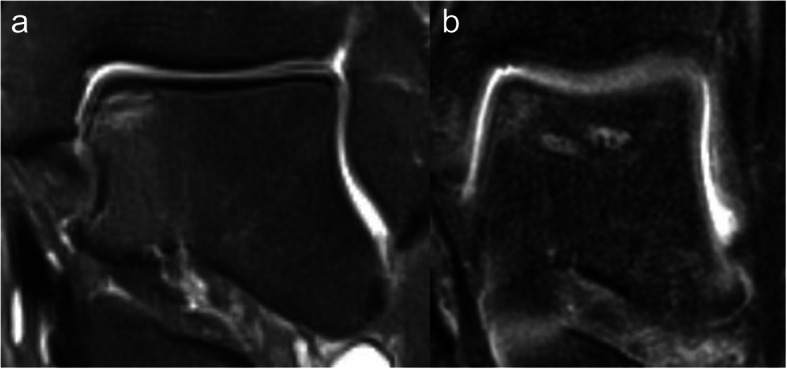
**a**/**b** MRI 6 months postoperatively after standard procedure with visual drilling channels and 2 years postoperatively with good bone union

**Fig. 4:**
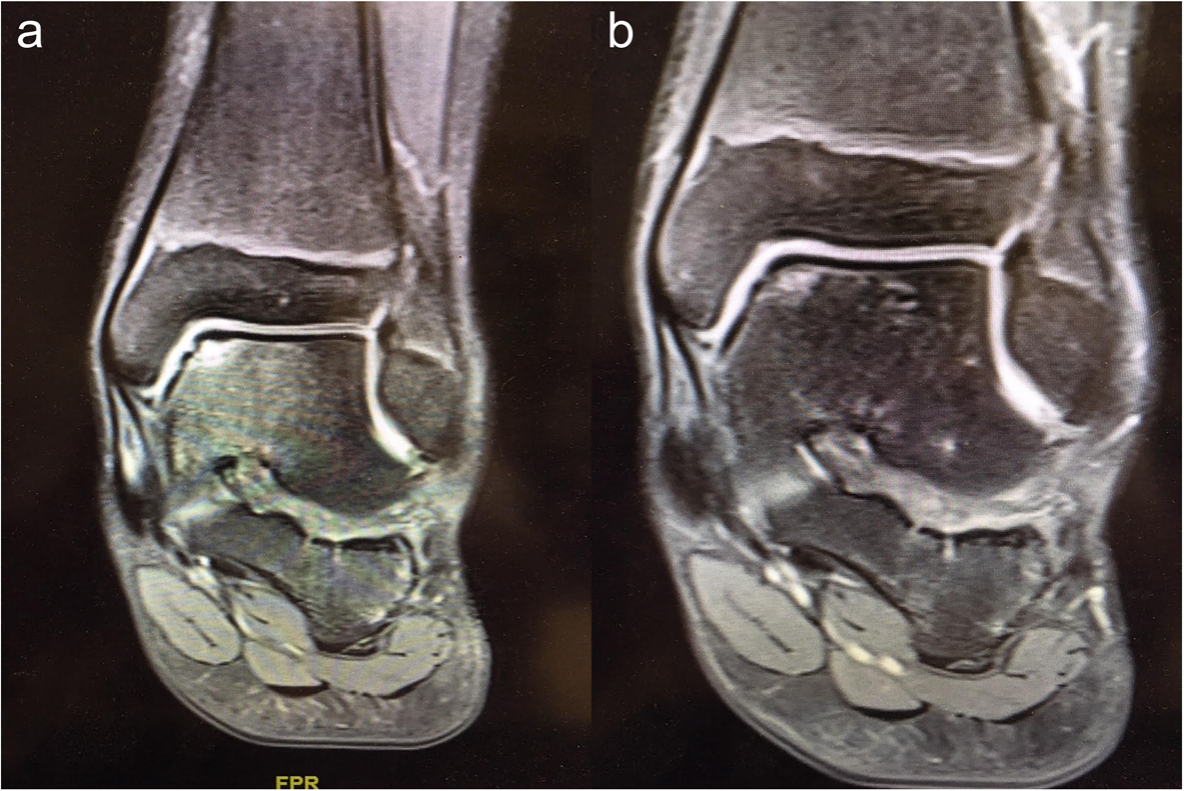
**a**/**b** MRI preoperatively (**a**) and 12 months postoperatively (**b**) in a 12 year old boy treated with retrograde drilling via electromagnetical guidance

Two clinical cases are presented in Fig. 3 (a/b) and Fig. 4 (a/b).

Clinically, all patients manifested full range of motion in the affected tibiotalar joint six months postoperatively in comparison to the unaffected side and were pain-free (VAS = 0) for activities of daily life. Swimming and cycling were possible in all cases, other sports were not permitted during the healing process.

## Discussion

This study demonstrates the superiority of the ENS method compared to the standard fluoroscopic technique with respect to reduction of length of surgery and radiation exposure. For the ENS method, no relevant setup or calibration times are necessary. An extra-operative site for the positioning of a stationary-based reference point is also not necessary. In contrast to two-dimensional fluoroscopic imaging, no switching between planes is required, so that drilling procedures can be shortened and the cumulative x-ray radiation exposure for both the patient and surgeon is significantly reduced [23].

Computer-assisted surgery for retrograde drilling procedures in OCD lesions has increased the precision of drilling [18, 19]. Navigation systems offer higher precision compared with fluoroscopy-guided techniques; thus, different navigation techniques have been established, such as opto-electronic guidance systems [21], 3-dimensional fluoroscopy–based [22], MRI-based [18], or computed tomography–based methods [17]. Good results have been published with computer-assisted minimally invasive retrograde drilling [11]. Nevertheless, navigated procedures are time-consuming operations as the setup, image acquisition, registration and verification must be obtained [24]. Furthermore, reference base–related complications such as iatrogenic fractures, heterotopic ossifications and unintended dislocation of the reference base have been documented [30].

To avoid drilling of the hyaline cartilage and its concomitant damage, retrograde drilling close to the subchondral bone with preservation of the cartilage surface is the favorable technique [9]. However, the complex anatomy of the ankle makes retrograde drilling challenging. Frail bone structure after repetitive attempts to reach the OCD lesion can result in iatrogenic fractures [25, 31]. Precise retrograde drilling without damaging the articular cartilage surface is thus very important [18, 32]. It is not always possible to use mechanical targeting devices, similar to those used for tunnel positioning in anterior cruciate ligament reconstruction [18], because their pre-shaped design does not always allow for accurate placement [10, 17].

In this study, an innovative ENS method with a small blunt probe hook, which serves as a dynamic target point, is presented. Additionally, a free choice of the starting point for the retrograde drilling with respect to important anatomic structures is possible, because the ENS method requires no pre-defined targeting angle to the tip of the probe hook.

Freehand fluoroscopy requires an average of approximately seven direction readjustments per operation, including backward drilling or even complete restarts [23]. Because real-time drilling direction information can be obtained on the monitor with the novel ENS method, readjustments of the drilling direction can be addressed concurrently within the drilling procedure. Regarding the drilling process time and radiation exposure, retrograde drilling with the ENS method required significant less operation time and radiation exposure. Furthermore, fluoroscopic controls were only used in this first clinical trial to make sure that the probe hook was in the correct position. In the future, fluoroscopic controls will not be needed at all.

Regarding financial resources in trauma care, introducing a new navigation system is always associated with acquisition costs. Benefits for all stakeholders have to be clear and visible [33]. However, length of surgery is an expense factor and a significant reduction leads to an increased cost-effectiveness overall as well as to lower infection rates.

The novel ENS method provided a time benefit of approximately 16min, which represents > 50 % of total operation time compared to the SFF method and a reduction in radiation exposure of approximately 40mSv per procedure.

Drilling of an OCD lesion of the distal femur has shown to have complete bone healing in up to 90 % of patients on an average after 6 months postoperatively [34]. Comparative literature for the talus is lacking. In a case series of six patients, Mosquijo et al. observed a relief of symptoms in all patients, but only three patients (50 %) showed complete healing on radiographs on a mean follow-up of 37 months [32]. Our clinical experience manifests a slow but continuous healing process of talar OCD lesions on follow-up MRIs.

### Limitations

Limitations of this study are the absence of power analysis and the low sample size in our single center study. While the precision of the drilling with the ENS method was well documented in previous cadaver studies. [16, 23–25] Our study focusses on the comparison of two operation methods and with this study design no conclusions can be made about precision of the drillings.

## Conclusions

In conclusion, the ENS method used in this study led to a reduction in length of surgery and required less x-ray radiation compared to the standard fluoroscopic technique. Cartilage perforation did not occur with this technique.

## Data Availability

The datasets used and analyzed during the current study are available from ODJ on reasonable request.
